# Successful Hair Regrowth in Early Frontal Fibrosing Alopecia With Combination Therapy

**DOI:** 10.7759/cureus.104784

**Published:** 2026-03-06

**Authors:** Sultana Kadasa, Mohammed Alturkistani, Abdulellah I Aleissa, Adel Alsantli

**Affiliations:** 1 College of Medicine, University of Jeddah, Jeddah, SAU; 2 College of Medicine, Umm Al-Qura University, Makkah, SAU; 3 Dermatology, King Fahad Armed Forces Hospital, Jeddah, SAU

**Keywords:** dutasteride, frontal fibrosing alopecia, multimodal therapy, scarring alopecia regrowth, tofacitinib

## Abstract

Frontal fibrosing alopecia (FFA) is a primary lymphocytic cicatricial alopecia that commonly presents with progressive frontotemporal hairline recession and eyebrow loss. Effective treatment remains challenging. We describe a 36-year-old woman with active postpartum-onset FFA who demonstrated significant improvement following combination therapy, including dutasteride, tofacitinib, and intralesional triamcinolone acetonide. This case highlights the potential benefit of early multimodal therapy in promoting regrowth in FFA, a condition typically associated with irreversible follicular loss.

## Introduction

Frontal fibrosing alopecia (FFA) is a primary cicatricial alopecia first described by Kossard in 1994 [1]. It is regarded as a variant of lichen planopilaris and typically presents with symmetric frontotemporal recession and loss of follicular openings [2,3]. Although it predominantly affects postmenopausal women, an increasing number of cases in premenopausal individuals suggests potential hormonal, autoimmune, or environmental triggers [2,4]. Dermoscopy commonly reveals perifollicular erythema, hyperkeratosis, and absent follicular ostia, features that assist in identifying active disease [3].

The global incidence of FFA appears to be rising, leading some authors to describe it as an “epidemic” of hair loss [2,4]. Eyebrow involvement is frequent, occurring in up to 73% of patients in some cohorts [2], and additional sites such as the occipital scalp, limbs, and axillae may also be affected [2,3].

Although the exact etiology remains unclear, hormonal influences are strongly suspected. Reports of disease onset following pregnancy or lactation suggest that hormonal fluctuations may contribute to the development of FFA [3]. Our patient’s postpartum onset aligns with this hypothesis. Additionally, although hormonal intrauterine devices (IUDs) have been studied, their role remains uncertain; one case-control study even reported a reduced risk of FFA among hormonal IUD users [5]. Autoimmune mechanisms have also been proposed, given histologic similarities to lichen planopilaris and associations with other autoimmune conditions in select patients [4]. A genetic predisposition is also possible, with up to 8% of patients reporting an affected family member [5].

Given the scarring and progressive nature of FFA, timely identification and intervention are essential. In this report, we describe a case of postpartum-onset FFA in a young woman who demonstrated significant clinical improvement following early, aggressive multimodal therapy.

## Case presentation

A 36-year-old woman presented with a two-year history of progressive frontotemporal hairline recession and partial eyebrow thinning. Symptoms began two years postpartum. She denied pain, pruritus, or scaling. Her medical history was unremarkable. She was using a hormonal IUD.

Physical examination revealed symmetric recession of the frontotemporal hairline, perifollicular erythema, scaling, and partial eyebrow loss. Dermoscopy demonstrated loss of follicular openings, blue-gray dots, and perifollicular hyperkeratosis.

Laboratory tests showed low ferritin (15 μg/L) and reduced transferrin saturation, with all other results normal. The laboratory findings are summarized in Table 1.

**Table 1:** Summary of laboratory findings with corresponding reference ranges. WBC, white blood cell count; Hb, hemoglobin; Hct, hematocrit; RBC, red blood cell count; MCV, mean corpuscular volume; MCH, mean corpuscular hemoglobin; RDW, red cell distribution width; TSH, thyroid-stimulating hormone; ALT, alanine aminotransferase; AST, aspartate aminotransferase.

Test	Result	Reference Range
White blood cell count (×10⁹/L)	10.5	3.3-10.8
Hemoglobin (g/L)	139	120-160
Hematocrit (L/L)	0.428	0.36-0.46
Red blood cell count (×10¹²/L)	5.12	4.0-5.2
Platelet count (×10⁹/L)	328-363	150-450
MCV (fL)	82.7	80-100
MCH (pg)	26.3	23.7-32
RDW (%)	14.4	11.8-15.5
Ferritin (µg/L)	15	15-204
Creatinine (µmol/L)	74	50-98
Vitamin B12 (pmol/L)	413	138-652
Folate (nmol/L)	13	7-46
TSH (mIU/L)	3.25	0.35-4.94
Transferrin saturation (%)	17	20-50
Total cholesterol (mmol/L)	3.9	≤5.2
LDL cholesterol (mmol/L)	2.4	≤2.6
HDL cholesterol (mmol/L)	1.4	≥1.3
Triglycerides (mmol/L)	0.7	≤1.7
ALT (U/L)	14	8-43
AST (U/L)	17	7-45
HbA1c (%)	5.1	4-5.6
Hepatitis B core antibody	Negative	Negative
Hepatitis C antibody	Negative	Negative
HIV 1 and 2	Negative	Negative

**Figure 1:**
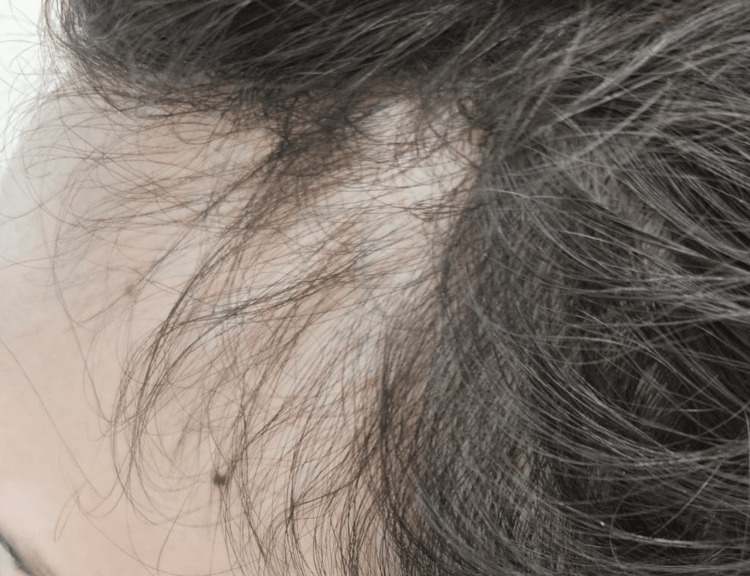
Post-treatment view of the left frontotemporal scalp Post-treatment improvement in hair density along the left frontotemporal scalp after six months of therapy.

**Figure 2:**
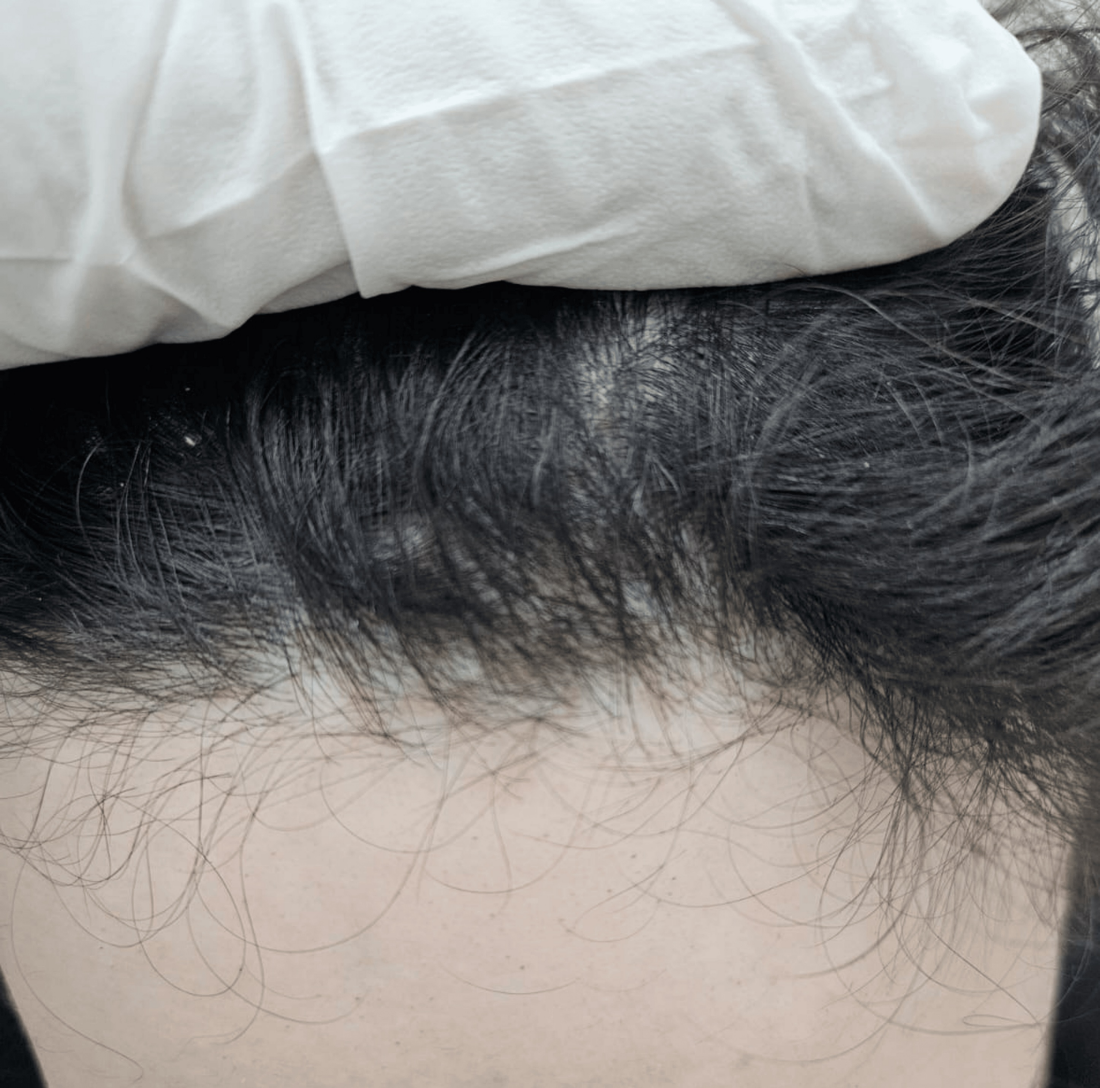
Post-treatment view of the frontotemporal hairline Post-treatment improvement in hair density along the frontotemporal scalp after six months of therapy.

**Figure 3:**
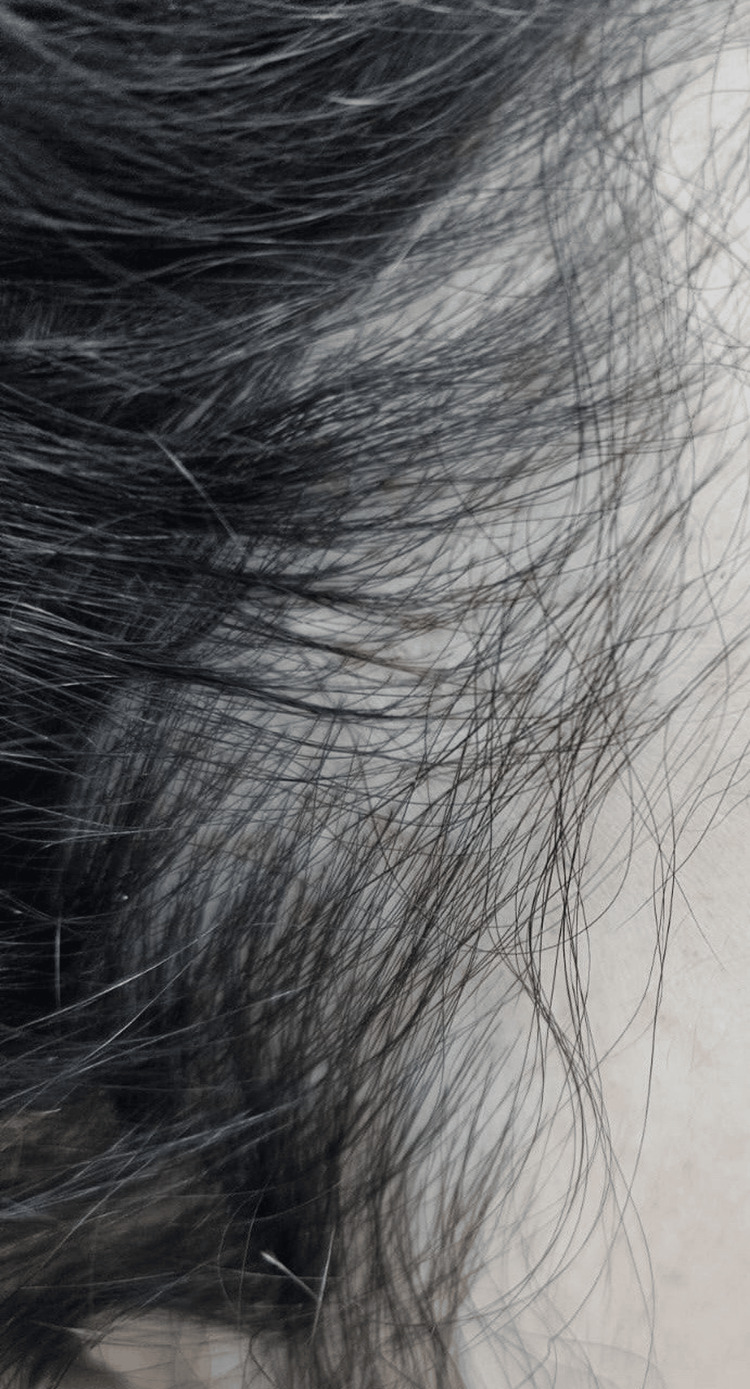
Post-treatment view of the right frontotemporal scalp Post-treatment improvement in hair density along the right frontotemporal scalp after six months of therapy.

Treatment included oral ferrous sulfate, dutasteride 0.5 mg daily, and, later, tofacitinib 10 mg twice daily for two months. She also received three sessions of intralesional triamcinolone acetonide (ILTA). After six months, she demonstrated marked regrowth of the frontotemporal hairline and eyebrows. Baseline clinical images demonstrate frontotemporal hairline recession with preserved vellus hairs, while post-treatment photographs show significant improvement in hair density along both the left and right frontotemporal scalp following six months of combination therapy (Figures 1-3).

## Discussion

FFA is a chronic lymphocytic scarring alopecia that can lead to irreversible follicular loss if not recognized and treated early. Although historically described in postmenopausal women, an increasing number of cases have been reported in premenopausal patients, suggesting that hormonal and environmental factors may contribute to disease onset [1-4]. Our patient’s postpartum onset supports previous theories that hormonal fluctuations may act as potential triggers [2]. The concurrent use of a hormonal IUD further highlights the complex endocrine interplay, as some reports suggest that hormonal IUDs may be associated with either increased or decreased risk of FFA [5]. Clinically, our patient exhibited classic features of active FFA, including symmetric frontotemporal hairline recession, perifollicular erythema, hyperkeratosis, absence of follicular openings, and eyebrow thinning, all well-described in the literature [1,2,6]. Differentiating FFA from postpartum telogen effluvium was essential. Telogen effluvium typically resolves within 6-12 months postpartum and does not present with scarring or perifollicular inflammatory signs [7]. The absence of mucosal or nail involvement and a negative autoimmune evaluation further supported FFA over other lichenoid disorders. Management of FFA is challenging due to the lack of randomized controlled trials and the variable disease course. Therapeutic strategies aim to reduce inflammation and halt progression. ILTA is considered a first-line therapy for active disease, especially in patients with perifollicular erythema or hyperkeratosis. Multiple retrospective studies demonstrate stabilization or improvement in more than 80% of patients when ILTA is used as part of combination therapy [2]. ILTA was used in our patient and likely contributed to the control of perifollicular inflammation. Systemic 5α-reductase inhibitors, such as finasteride and dutasteride, have demonstrated some of the strongest evidence for disease stabilization in FFA. Finasteride has been reported to stabilize or improve disease in up to 100% of patients in one cohort [2], while dutasteride may offer superior efficacy due to inhibition of both type I and II 5α-reductase isoenzymes. Dutasteride 0.5 mg daily is widely used and supported by multiple case series showing reduced progression and, in some cases, regrowth [1-3]. Because of their teratogenic potential, these agents require reliable contraception in premenopausal women. The patient’s hormonal IUD provided adequate protection, consistent with current safety recommendations.

Tofacitinib, a Janus kinase (JAK) inhibitor, was added to the regimen because of the progressive nature of the disease. Although not yet a standard therapy for FFA, emerging case reports have shown promising results in refractory scarring alopecias, including visible regrowth within 6-9 months of treatment [2]. JAK inhibitors may be beneficial by downregulating inflammatory cytokine pathways implicated in the lichenoid damage characteristic of FFA. Our patient demonstrated significant improvement after the introduction of tofacitinib, consistent with these early reports. Iron deficiency, identified by low ferritin and transferrin saturation, was also addressed. Although iron deficiency is more strongly associated with telogen effluvium, optimizing ferritin levels may improve overall hair health and complement other therapies, even though iron repletion alone is unlikely to explain the degree of regrowth observed. Additional therapies described in the literature include hydroxychloroquine, retinoids, tetracyclines, and pioglitazone, each with variable success rates [2,4]. Combination therapy is generally more effective than monotherapy. Hair transplantation is reserved for stable disease, but recurrence is common if residual inflammation persists [4]. The significant hair regrowth observed in this patient is notable, as FFA is typically considered a scarring alopecia with limited potential for follicular recovery once fibrosis has developed. This case supports the concept that early diagnosis and aggressive multimodal therapy, targeting both hormonal and inflammatory mechanisms, may preserve partially damaged follicles and allow clinically meaningful regrowth. Overall, this case highlights the importance of recognizing FFA in younger women, understanding potential hormonal influences, and considering a comprehensive treatment strategy, including dutasteride, JAK inhibition, and ILTA, to optimize outcomes in this challenging condition.

## Conclusions

FFA can occur in premenopausal women and may be influenced by hormonal changes. Early diagnosis and multimodal therapy, including dutasteride, JAK inhibitors, and intralesional corticosteroids, may help achieve stabilization and regrowth. Continued monitoring is essential to ensure long-term control.
